# A cross-sectional study of non-invasive markers of vascular health in children and adolescents

**DOI:** 10.1038/s41390-025-04041-w

**Published:** 2025-05-03

**Authors:** Reeja F. Nasir, Tommy Y. Cai, Alice Meroni, Hasthi Dissanayake, Adrienne Gordon, David S. Celermajer, Michael R. Skilton

**Affiliations:** 1 The Boden Initiative, Charles Perkins Centre, University of Sydney, Camperdown, NSW Australia; 2 Faculty of Medicine and Health, University of Sydney, Camperdown, NSW Australia; 3 Department of Vascular Surgery, Royal North Shore Hospital, Northern Sydney Local Health District, Sydney, NSW Australia; 4 Department of Obstetrics, Gynaecology and Neonatology, Royal Prince Alfred Hospital, Sydney Local Health District, Sydney, NSW Australia; 5 Sydney Institute for Women, Children and their Families, Sydney Local Health District, Sydney, NSW Australia; 6 Department of Cardiology, Royal Prince Alfred Hospital, Sydney Local Health District, Sydney, NSW Australia

## Abstract

**Background:**

Non-invasive methods assessing subclinical atherosclerosis, such as carotid intima-media thickness (IMT) and carotid-femoral pulse wave velocity (cfPWV), are well-described in adults but less so in the paediatric population. Other techniques, such as aortic IMT, may be more appropriate in children. This study aimed to characterise age-related changes to arterial vasculature using best practice methodology for these measures.

**Methods:**

This cross-sectional study involved 97 healthy volunteers aged 2 to 20 (mean age 11.2 years [SD 5.1]). Carotid and aortic IMT were measured using high-resolution ultrasound. A validated semi-automated cuff-based device was used to measure cfPWV.

**Results:**

Aortic IMT showed a significant age-related increase of 9 μm per year [95% CI: 6, 12], *p* < 0.0001) during childhood and adolescence, whilst carotid IMT was significantly associated with male sex (29 μm per year [95% CI: 11, 48], *p* = 0.002) during this period. cfPWV exhibited an age-related increase from nine years of age onwards (0.11 ms^-1^ per year [95% CI: 0.06, 0.15], *p* < 0.001).

**Conclusions:**

Age-related changes in the arterial vasculature vary between sites and methodologies. During childhood, aortic IMT appears to be a more sensitive marker for early structural changes and should be prioritised over carotid IMT and cfPWV.

**Impact:**

- Understanding age-related changes in arterial vasculature via non-invasive measures is poorly described in childhood and adolescence.
- This study finds that the timing and magnitude of age-related changes vary across arterial beds and between non-invasive measures in the first two decades of life.
- Aortic intima-media thickness is a more age-appropriate measure of early structural changes in the young than the more widely used carotid intima-media thickness.
- Furthermore, carotid-femoral pulse wave velocity, a measure of aortic stiffness, is best evaluated in late childhood and adolescence and likely has little relevance in early childhood.

## Introduction

Atherosclerosis is the gradual and chronic inflammatory disease underlying most clinical cardiovascular events. Non-invasive methods for assessing early arterial disease include intima-media thickness (cIMT) for measuring early structural changes and carotid-femoral pulse wave velocity (cfPWV) for measuring stiffness. These have been established and validated in older populations (≥ 45 years) and are predictive of future cardiovascular events,^1–3^ but the application of these methods in childhood and adolescence is less well described and standardised. Nonetheless, the use of non-invasive measures as surrogates for clinical outcomes is necessitated during this period to better identify and characterise early life risk factors for cardiovascular disease.

The above measures are commonly applied in infants and young children; however, postmortem studies on the natural history of atherosclerosis suggest that the IMT of the abdominal aorta (aIMT) may be a more sensitive marker for structural changes consistent with the earliest physical manifestations of atherosclerosis. The earliest lesions manifest in the abdominal aorta during the first decade of life,^3^ whereas they do not appear in the common carotid artery until mid-childhood.^4,5^ The extent and severity of these early aortic lesions in childhood are associated with early life risk factors for CVD,^6,7^ and their localisation is consistent with more complex and occlusive lesions later in life.^4^ Similarly, the aorta appears to have some capacity to buffer against haemodynamic stress in childhood before increasingly stiffening with age.^8^

Understanding age-related changes in the arteries during the first two decades of life is an essential prelude for interpreting studies that apply surrogate markers in at-risk children. Prior studies have reported non-invasive measures of vascular health in a narrow age range, and differences in methodologies make comparison across studies difficult. Accordingly, we sought to characterise age-related changes in arterial structure during childhood and adolescence, using the best-practice methodology of established and emerging surrogate markers, in a group of healthy volunteers aged two to twenty.

## Patients and methods

### Participants

A total of 97 healthy volunteers were prospectively recruited between August 2016 and January 2019 from the University of Sydney, Royal Prince Alfred Hospital, and surrounding localities (Sydney, Australia) for a cross-sectional study investigating central blood pressure in children and adolescents (2-to-20 Children’s Cardiovascular Health Study). Recruitment was conducted via posters, social media, and word-of-mouth. Participants were purposively recruited across five equally distributed age groups, with near-equal sex representation, between the ages of 2 and 20. This was to prevent an unequal age distribution across the broad age range. These groups were defined as early childhood: 2–6.5 years (*n* = 20); middle childhood 1: 6.6–9.5 years (*n* = 20); middle childhood 2: 9.6–12.5 years (*n* = 20); early adolescence: 12.6–15.5 years (*n* = 17); late adolescence: 15.6–20 (*n* = 20). There were no strict exclusion criteria. Study visits were undertaken at the Charles Perkins Centre Royal Prince Alfred Clinic.

This study was conducted in accordance with the Declaration of Helsinki and approved by the Human Research Ethics Committee of the Sydney Local Health District (Protocol No. X16-0065 and HREC/16/RPAH/18). Participation was voluntary, and informed consent was obtained from each participant or their parent for those under 18.

### Anthropometrics

Body weight was measured using a digital scale (BC-418 Segmental Body Composition Analyzer, Tanita Australia, Kewdale, WA, Australia), with participants wearing light clothing and no shoes. Standing height was measured barefoot using a wall-mounted stadiometer (HR-200 Wall-mounted Height Rod, Tanita Australia). BMI was calculated as body mass (kg)/height (m)^2^. Anthropometric z-scores were calculated using the Lambda-Mu-Sigma method detailed in the Centres for Disease Control and Prevention 2000 Growth Charts.^9^ Overweight and  obesity were categorised as BMI z-scores of > + 1 SD and > + 2 SD, respectively.

### Carotid-femoral pulse wave velocity and blood pressure

cfPWV was measured as per Artery Society guidelines^10,11^ using an automated cuff-based device. This involves measuring the carotid pulse waveform with a tonometer, whilst the femoral waveform is detected simultaneously by volume displacement produced by a cuff inflated around the upper thigh. The arterial path between carotid and femoral pulses is estimated by subtracting the distance between the sternal notch and the carotid pulse from the distance between the sternal notch and the femoral pulse. These distances are measured using a non-stretchable tape, independent of body habitus. This method is shown to have an acceptable agreement with the tonometric method, the current recommended reference standard in place of an invasive measurement.^12^ In the tonometric method, the carotid and femoral waveforms are recorded sequentially with a tonometer using electrocardiogram-gating.^12^ A detailed description of the methodology and validation of cuff-based assessment of cfPWV in a subset of study participants is described elsewhere.^13^

All assessments were performed in a quiet room, with the participant resting in a supine position for at least 15 min beforehand. Measurements were taken in triplicate with the SphygmoCor XCEL System (Version 9; AtCor Medical Systems, Sydney, NSW, Australia) as per the manufacturer’s protocol by a trained operator (A.M.). The raw PWV values were then transformed with a paediatric transfer function as described by Cai et al. (2020)^13^ and averaged to derive cfPWV. Brachial blood pressure was measured three times using the SphygmoCor XCEL System with an appropriately sized cuff. Mean arterial pressure (MAP) was calculated as DP + 1/3(SP – DP).

### Aortic and carotid intima-media thickness

The methodologies for assessing aortic and cIMT followed best-practice guidelines.^3,14,15^ Due to feasibility concerns in young children, cIMT was assessed only in participants aged six years and older, whilst aIMT was assessed in all participants.^3^ Images were captured using high-resolution B-mode ultrasonography (EPIQ 7; Philips Medical Systems, Best, NB, Netherlands) with a high-frequency linear array probe (12–3 MHz) in a quiet, temperature-controlled darkened room with the participant in a supine position without a pillow. Ultrasound settings were standardised using a customised preset function, and a three-lead electrocardiogram (ECG) was collected simultaneously. Depth and zoom were adjusted to accommodate changes in body size. A single experienced sonographer performed both assessments (A.M.). For aIMT, a longitudinal straight, non-branched segment of the abdominal aorta was imaged between the xiphisternum and umbilicus. For cIMT, bilateral examination of the common carotid arteries was performed proximal to the carotid bulb with three pre-specified angles of insonation per side using a Meijer’s Carotid Arc (Meijer’s Medical Ultrasound, Voorschoten, ZH, Netherlands) for standardisation and reproducibility.^15^

Images underwent subsequent blinded off-line analysis using a validated semi-automated edge-detection software (Carotid Analyzer, Version 5; Medical Imaging Applications LLC, Coralville, IA) by experienced observers (T.Y.C. and R.N.), with manual tracing adjustment when required. Far-wall mean and maximum IMT and minimum vessel diameter were measured from a straight region of interest (ROI) in three cardiac cycles at the end of diastole (on or closest to the R-wave of the ECG). For the aorta, the ROI was a non-branched segment between the proximal aorta and iliac bifurcation, with a minimum 4 mm distance. For the carotid, the ROI was positioned a maximum of 5 mm distal to the carotid bifurcation, with a minimum 10 mm distance. IMT was measured per angle and averaged to produce the mean and maximum cIMT. Maximum IMT was our primary outcome, given the focal nature of early arterial injury in the young.^16^ To account for the physiological growth of the vessel, we tested minimum vessel diameter as a covariate in regression analyses of IMT and the IMT-to-lumen ratio (IMT divided by minimum vessel diameter) as a dependent variable.^17,18^ A number of scans (*n* = 20) analysed by T.Y.C. were randomly selected and re-analysed by R.N. to calculate the interobserver reliability. The intraclass correlation coefficient for maximum IMT was 0.90 (95% CI: 0.78, 0.96), indicating “excellent” reliability.^19,20^

### Statistical analysis

Descriptive data are presented as means (SDs) for continuous variables and n (%) for categorical variables. Data was visually assessed for normality distribution and confirmed using the Kolmogorov-Smirnov test. Linear associations were determined using Pearson’s correlation and multivariable linear regression. Multivariable linear regression was adjusted for age, sex, height z-score, and minimum vessel diameter. Variable selection was hypothesis-driven. An interaction term (*p*_Interaction_) was used to determine whether associations of vascular measures with age differed by sex. Statistical significance was inferred with a two-sided *p* < 0.05. For cfPWV, we conducted segmented (piecewise) regression analysis using RStudio (Version 4.4.1, R Core Team 2024). Firstly, the “segmented” package^21^ was used to identify an inflection point (i.e., age) where the relationship between age and cfPWV changed. Then, segmented linear regression was done to estimate regression coefficients between age and cfPWV for participants with ages above and below this inflection point, adjusted for MAP. Statistical analyses were performed using SPSS (Version 26.0; IBM Corporation, Armonk, NY) and RStudio. Figures were plotted in GraphPad Prism (Version 8; GraphPad Software, Inc., San Diego, CA).

## Results

Participant characteristics are summarised in Table 1. The mean age of this cohort was 11.2 ± 5.1 SD years. Two participants were classified as having obesity, and eight participants were classified as having overweight at presentation. All participants were non-diabetic, non-hypertensive, and had never smoked. Of the 97 participants, aIMT images were collected from 87. The most common reasons for being unable to image the aorta were participant unrest and sonographic artifacts from gas in the bowel. An additional seven participants had ultrasound images of insufficient quality for IMT analysis, i.e., we were unable to measure a minimum 4 mm straight segment or obtain a measurement from a minimum of three cardiac cycles at end-diastole. Weight z-score was significantly greater (*p* = 0.02) in those without an aIMT measurement than those with (Supplementary Table S1). cIMT was successfully collected and measured from all 75 participants aged 6–20. cfPWV was successfully collected from all participants; however, one participant was excluded from the analysis due to an implausible transit distance being recorded.

**Table 1:** Participant characteristics, aortic and carotid intima-media thickness, and carotid-femoral pulse wave velocity for all participants and by recruitment group.

	All (*N* = 97)	Early childhood (2–6.5 years; *n* = 20)	Middle Childhood 1 (6.6–9.5 years; *n* = 20)	Middle childhood 2 (9.6–12.5 years; *n* = 20)	Early adolescence (12.6–15.5 years; *n* = 17)	Late adolescence (15.6–20 years; *n* = 20)
*Characteristics*						
Age (years)	11.2 (5.1)	4.3 (0.9)	8.2 (0.8)	11.0 (1.0)	14.2 (0.9)	18.8 (1.5)
Sex (Female)	49 (50.5)	10 (50.0)	10 (50.0)	10 (50.0)	9 (52.9)	10 (50.0)
Weight (kg)	39.5 (19.0)	17.3 (2.4)	28.2 (4.3)	38.0 (7.7)	50.6 (6.4)	65.2 (15.7)
Weight z-score	0.11 (0.94)	0.14 (0.97)	0.39 (0.84)	0.02 (0.90)	−0.05 (0.70)	−0.01 (1.20)
Height (cm)	143.1 (24.6)	106.3 (7.2)	132.0 (7.6)	146.6 (8.0)	163.4 (7.4)	170.4 (11.1)
Height z-score	0.16 (2.56)	0.58 (0.84)	0.58 (1.00)	0.32 (1.00)	0.29 (1.09)	0.18 (1.17)
BMI (kg/m^2^)	18.0 (3.3)	15.3 (1.7)	16.2 (1.8)	17.6 (2.8)	18.9 (1.6)	22.1 (3.0)
BMI z-score	-0.09 (0.93)	−0.20 (1.14)	0.11 (0.82)	−0.08 (1.01)	−0.24 (0.62)	−0.05 (0.99)
*Intima-media Thickness*						
Maximum aIMT (μm)^a^	630 (104)	532 (98)	611 (70)	632 (104)	666 (90)	713 (77)
Mean aIMT (μm)^a^	541 (83)	465 (99)	528 (57)	541 (76)	577 (66)	595 (57)
Aortic lumen diameter (μm)	9218 (2189)	6145 (1054)	8238 (966)	9220 (1329)	10373 (1000)	11439 (1951)
Maximum aIMT-to-lumen ratio^b^	0.07 (0.01)	0.09 (0.02)	0.07 (0.01)	0.07 (0.01)	0.07 (0.01)	0.06 (0.01)
Mean aIMT-to-lumen ratio^b^	0.06 (0.01)	0.08 (0.02)	0.06 (0.01)	0.06 (0.01)	0.06 (0.01)	0.05 (0.01)
Maximum cIMT (μm)^c^	558 (43)	-	546 (40)	557 (34)	563 (42)	568 (52)
Mean cIMT (μm)^c^	480 (48)	-	463 (48)	486 (46)	485 (48)	487 (50)
Carotid lumen diameter (μm)	6030 (443)	-	5854 (328)	5964 (391)	5939 (371)	6338 (507)
Maximum cIMT-to-lumen ratio^c^	0.09 (0.01)	-	0.09 (0.01)	0.09 (0.01)	0.09 (0.01)	0.09 (0.01)
Mean cIMT-to-lumen ratio^c^	0.08 (0.01)	-	0.08 (0.01)	0.08 (0.01)	0.08 (0.01)	0.08 (0.01)
*Pulse Wave Velocity*						
cfPWV (ms^-1^)^d^	4.55 (0.72)	4.21 (0.70)	4.19 (0.33)	4.33 (0.55)	4.83 (0.43)	5.26 (0.79)

Values are mean (SD) for continuous variables or [n (%)] for categorical variables.

*BMI* body mass index, *aIMT* aortic intima-media thickness, *cIMT* carotid intima-media thickness, *cfPWV* carotid-femoral pulse wave velocity.

^a^Participant numbers for aIMT; Overall (*N* = 80), Early childhood (*n* = 15), Middle childhood 1 (*n* = 19), Middle childhood 2 (*n* = 16), Early adolescence (*n* = 16) and Late adolescence (*n* = 15).

^b^Participant numbers for aIMT-to-lumen ratio; Overall (*N* = 77), Early childhood (*n* = 13), Middle childhood 1 (*n* = 19), Middle childhood 2 (*n* = 15), Early adolescence (*n* = 15) and Late adolescence (*n* = 15).

^c^Participant numbers for cIMT and cIMT-to-lumen ratio; Overall (*N* = 75), Middle childhood 1 (*n* = 19), Middle childhood 2 (*n* = 20), Group 4 (*n* = 16) and Group 5 (*n* = 20).

^d^Participant numbers for cfPWV; Overall (*N* = 96), Early childhood (*n* = 20), Middle childhood 1 (*n* = 20), Middle childhood 2 (*n* = 20), Early adolescence (*n* = 16) and Late adolescence (*n* = 20).

### Intima-media thickness and IMT-to-lumen ratio

**Fig. 1:**
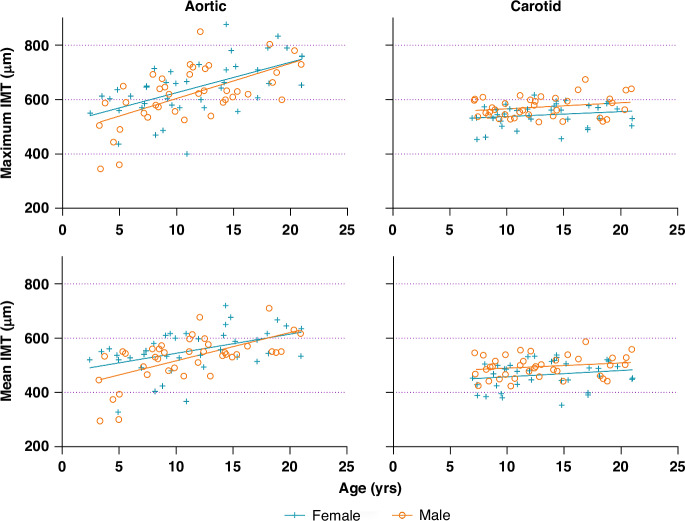
Scatter plot displaying the association of age with maximum and mean aortic intima-media thickness (aIMT) and carotid intima-media thickness (cIMT) for healthy males and females during childhood and adolescence. aIMT was assessed in children aged 2–20; cIMT was assessed in the subgroup of children aged 6.5–20.

Maximum and mean aIMT significantly increased with age (maximum aIMT 12 μm per year [95% CI: 8, 16), *p* < 0.0001, R^2^ = 0.33; mean aIMT 9 μm per year [95% CI: 6, 12], *p* < 0.0001, R^2^ = 0.27). These remained significant after adjustment for sex, height z-score, and minimum vessel diameter (maximum aIMT *p* = 0.02, R^2^ = 0.35; mean aIMT *p* = 0.03, R^2^ = 0. 31). Sex was not significantly associated with aIMT (maximum aIMT *p* = 0.17, mean aIMT *p* = 0.35) and we did not find any evidence of interaction by sex (maximum aIMT *p*_Interaction_ = 0.31; mean aIMT *p*_Interaction_ = 0.98). Univariate associations of age with aortic or cIMT are shown in Fig. 1.

Maximum and mean cIMT did increase with age; however, this was not significant in either univariate analysis (maximum cIMT 2 μm per year [95% CI: −0, 4], *p* = 0.07, R^2^ = 0.02; mean cIMT 2 μm per year [95% CI: −1, 5], *p* = 0.14, R^2^ = 0.02) or analysis adjusting for sex, height z-score and minimum vessel diameter (maximum cIMT *p* = 0.23, R^2^ = 0.19; mean cIMT *p* = 0.62, R^2^ = 0.19). However, sex was strongly associated with cIMT (sex [male]: maximum cIMT 30 μm per year [95% CI 9, 51], *p* = 0.005, R^2^ = 0.09; mean cIMT 29 μm per year [95% CI: 11, 48], *p* = 0.002, R^2^ = 0.11). The association of sex with cIMT did not change when adjusted for age, height z-score, and minimum vessel diameter (maximum cIMT *p* = 0.005; mean cIMT *p* = 0.01).

Maximum IMT-to-lumen ratio significantly decreased with age in the aorta (−0.001 per year [95% CI −0.002, 0.000], *p* = 0.02, R^2^ = 0.24) whilst in the carotid it significantly increased with age (0.000 per year [95% CI −0.001, 0.000], *p* = 0.05, R^2^ = 0.02) (Supplementary Fig. S1). When adjusted for sex, male sex was significantly associated with decreased maximum IMT-to-lumen ratio in the aorta (−0.006 [95% CI: −0.001, −0.01], *p* = 0.03, R^2^ = 0.28). There was no evidence of interaction between age and sex in both vessels (maximum aIMT-to-lumen ratio *p*_Interaction_ = 0.79; maximum cIMT-to-lumen ratio *p*_Interaction_ = 0.09). Results were similar for mean IMT-to-lumen ratio and following additional adjustment for height z-score (data not shown).

### Carotid-femoral pulse wave velocity

**Fig. 2:**
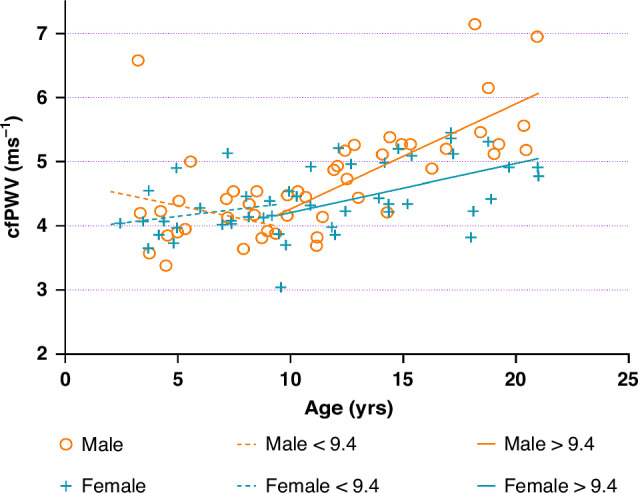
Scatter plot displaying the association of age with carotid-femoral pulse wave velocity (cfPWV) for healthy males and females during childhood and adolescence, aged 2–20. Overlying regression lines represent the association before and after an inflection point of 9.4.

We observed an apparent increase in cfPWV beginning at approximately 8–10 years of age (Fig. 2; Supplementary Fig. S2). Segmented regression analysis via RStudio identified an inflection point at 9.4 years of age. In those younger than 9.4 years, cfPWV was not associated with age (−0.00 ms^−1^ per year [95% CI: −0.09, 0.08], *p* = 0.95, R^2^ = 0.12), whereas in those older than 9.4 years of age, cfPWV significantly increased with age (0.11 ms^−1^ per year [95% CI: 0.06, 0.15], *p* < 0.001, R^2^ = 0.48). In the older group, there was evidence of sex-specific differences (*p*_Interaction_ = 0.00), with the association of age with cfPWV being markedly greater in males (0.14 ms^−1^ per year [95% CI: 0.07, 0.20], *p* < 0.0001, *n* = 27, R^2^ = 0.61) than in females (0.08 ms^−1^ per year [95% CI: 0.03, 0.13], *p* = 0.003, *n* = 28, R^2^ = 0.42). All regression analyses were investigated excluding those with overweight or obesity, and there was no substantial effect on the findings (data not shown).

## Discussion

In this cross-sectional study, we find that both aortic and carotid IMT increased linearly with age throughout childhood and adolescence, with aIMT showing a greater magnitude of increase. However, only aIMT showed a statistically significant relationship with age. During this period, cIMT is significantly associated with male sex. Additionally, cfPWV is not meaningfully associated with age until approximately nine years, after which there is a marked age-related increase, particularly in males. These findings suggest that a selection of age-appropriate methods is required for accurate, non-invasive assessment of vascular health during childhood and adolescence.

Our observations are consistent with evidence from postmortem studies, which show that lesions manifest earlier in life in the abdominal aorta than they do in the common carotid artery. Indeed, the earliest evidence of structural changes in the aorta is present in the fetus and throughout early childhood.^6,7,16^ In contrast, the same structural changes begin to develop in the common carotid arteries only from mid-childhood. In our study, we did not find evidence of an “age of onset” for carotid intima-media thickening; rather, we found that the age-associated increase in cIMT was less pronounced than that of aIMT. These divergent findings indicate there may be distinct differences in the pathophysiology of atherosclerosis between the carotid and aortic vascular beds. For this reason, we consider aIMT particularly advantageous, more so than cIMT, to study in relation to risk factors in younger populations, as its faster progression could provide an earlier or more sensitive indication of cardiovascular risk. Similar associations have previously been reported in a cohort of older adolescents and young adults^22^ in which both aIMT and cIMT were shown to increase with age; however, aIMT was proposed as a more sensitive marker of early vascular changes. Our results extend this concept to a younger age group, from early childhood to adolescence. While we did not investigate the underlying causes of these divergent findings between the two vascular beds, possible contributing factors could be differences in shear and tensile stress or blood pressure. This is supported by findings from Semmler et al. (2021)^23^, who found that differences in IMT, diameter, and their ratio in the common carotid between males and females are partly driven by changes in these hemodynamic parameters, which are influenced by puberty or excessive adiposity.

Some amount of IMT thickening is reactionary to an increase in vessel diameter.^17^ In adults, this may be in an intermediatory phenotype of cardiovascular disease [23]. However, in children, age-related vessel growth is expected.^3,18^ Recent findings from the Avon Longitudinal Study of Parents and Children showed that lean mass was the strongest determinant of increased carotid diameter in young adults aged 17−21. This was accompanied by a proportional increase in cIMT, driven by an increase in the medial layer — a non-atherosclerotic process — which resulted in a preserved IMT-to-lumen ratio with age.^24^ Conversely, exposure to excessive adiposity increased diameter without a proportional increase in cIMT, leading to a reduced cIMT-to-lumen ratio and increased circumferential wall stress.

Our study explored two methods to account for the normal physiological relationship between IMT and vessel size.^3,18^ First, we adjusted for minimum vessel diameter in our regression analyses, which did not significantly alter our findings with aIMT and age or cIMT and male sex. Secondly, we used the IMT-to-lumen ratio as the outcome measure. In this cohort, the aIMT-to-lumen ratio decreased with age, particularly in males, whereas the cIMT-to-lumen ratio showed a constant increase with age. Our results suggest that during the first two decades of life in the carotid, any increase in IMT is relatively proportionate to an increase in vessel size, thus representing growth-related remodelling. In contrast, in the abdominal aorta, there is evidence of remodelling that may be more sensitive to risk factors. We interpret this with caution due to our small sample size and the cross-sectional study design. Nonetheless, it is apparent that IMT alone cannot sufficiently explain vascular adaptations or pathological processes in younger individuals. Future studies are needed to identify the factors influencing diameter and IMT-to-lumen ratio in the abdominal aorta to better differentiate between growth-related remodelling and pathological changes.

We found that cfPWV, a measure of aortic stiffness, remained relatively unchanged through early childhood in both males and females. However, from approximately nine years of age onwards, there was a marked age-related increase in cfPWV, particularly in males. This is similar to the findings of Hidvegi et al. (2021), who found an age-related increase in cfPWV at twelve years in males and ten years in females after a period without a notable increase between 3 and 8 years of age.^25^ Given that age is one of the strongest risk factors for arterial stiffening, these results suggest that material changes in arterial stiffness do not occur in young children. As such, we caution against the assessment of cfPWV in this age group. This would also be consistent with the hypothesis that the arterial vasculature can functionally compensate for any structural changes that are consistent with stiffening during early childhood,^26,27^ until a threshold which in some may be around nine years of age. A possible mechanism for this may be the synthesis of elastin fibres in the media, which is shown to peak during childhood. After this age, age-related and mechanical processes cause gradual elastin degradation and increasing arterial stiffness. This pattern aligns with findings from Laogun and Gosling (1982),^8^ who found arterial compliance was lower at birth compared to 8−10 years of age and then steadily began to decrease till reaching birth values in a sex-dependent manner.

A key strength of this study is the application of best-practice methodologies across a wide age range, including early childhood. Previous studies have applied these methodologies to either very young or older adolescents, making direct comparisons difficult. Accordingly, our findings provide some indication of normative levels of aIMT, cIMT, IMT-to-lumen ratio, and cfPWV in healthy children and adolescents, albeit from a relatively small sample.

Limitations of our work include using a cross-sectional study design to infer changes across childhood and adolescence. A prospective cohort study with repeated 12-month follow-ups would be the ideal design to definitively profile age-related vascular changes using the methods described; however, due to the large number of participants, the time and cost involved, and the technical expertise required to conduct these assessments in children, such a study would be challenging to conduct.^28^ Additionally, the primary study was powered to detect changes in blood pressure; thus, this secondary analysis may be underpowered. This pragmatic study aimed to evaluate these measures within a non-selected, generally healthy population. Considering the high prevalence of overweight and obesity among Australian children and adolescents (25%), we sought to be as inclusive as possible and did not exclude this cohort. Instead, we aimed to conduct a sensitivity analysis to account for this. Our analysis was robust to sensitivity, and we did not find any differences in IMT, diameter, and IMT-to-lumen ratio between those with overweight or obesity and those without. However, we note that higher BMI in children has been previously shown to be positively associated with IMT and diameter and negatively associated with IMT-to-lumen ratio.^23^

Another limitation is that we measured aIMT from a single non-branched segment between the proximal aorta and the iliac bifurcation. The initial protocol prescribed measuring aIMT at multiple segments between the coeliac trunk and the iliac arteries, corresponding approximately to the upper, middle, and lower abdominal aorta. However, due to frequent participant movement and ultrasound artefact, this approach was not feasible for all participants. Therefore, the most optimal, clearly visualised segment between the xiphisternum and umbilicus was prioritised. It should also be noted that branching points in the abdominal aorta produce variations in shear stress, making certain regions more susceptible to atheroma formation; in particular, the dorsal wall of the distal abdominal aorta, just before the iliac bifurcation, is especially vulnerable to lesion development and progression.^3^ This limitation is unlikely to impact our findings as any potential errors or regional variations in lesion susceptibility should be averaged at a study population level, providing a robust overall assessment of age-related changes in the aorta. However, future studies examining aIMT responses to adverse exposures or interventions should standardise measurement sites or specify regions imaged, as inconsistent sites could obscure effects. Lastly, aIMT was successfully measured in only about 80% of participants, which is lower than the recommended threshold for missing data.^3^ Accurate visualisation of aIMT requires high scanning frequencies (approximately 10 MHz), which can limit its applicability in those with higher central adiposity.^3,22,29^ Indeed, we observed that the weight z-score was significantly greater in those without a successful aIMT measurement. Future studies may seek to identify whether using curved linear transducers might improve measurement success in subjects with higher BMI. Finally, due to varying ages, not all participants could comply with fasting overnight, but this has been noted to improve the visualisation of aIMT and should be applied in future studies.^22^

## Conclusion

In conclusion, non-invasive measures of structural vascular health, specifically aIMT, cIMT, and cfPWV, all progress in severity with age in the young; however, the timing and magnitude of these changes differ between arterial sites and methodologies. This indicates the importance of selecting age-appropriate methods for assessing vascular health in childhood and adolescence.

## Supplementary information

Supplementary Material

## Data Availability

The datasets analysed during the current study are not publicly available as the participants or their parent/guardian did not consent to them being publicly available, but they are available from the corresponding author upon reasonable request.
