# Supplementary material for: A cross-sectional study of non-invasive markers of vascular health in children and adolescents
Source: Pediatr Res. 2025 May 3;98(6):2394–400. doi: 10.1038/s41390-025-04041-w (PMC12811110; doi:10.1038/s41390-025-04041-w)
Supplement: Supplementary file 1 — Supplementary Material [file 41390_2025_4041_MOESM1_ESM.pdf]

Supplementary Material

Supplementary Table 1. Demographic characteristics by those with and without a successful aortic intima-media thickness measurement.

	With aIMT (n = 80)	Without aIMT (n = 17)	*p*-value
Characteristic			
Age (years)	11.2 (5.0)	11.1 (6.0)	0.95
Sex (% Female)	50.0	52.9	0.83
Weight (kg)	38.7 (18.2)	43.2 (22.9)	0.38
Weight z-score	0.00 (0.91)	0.60 (0.94)	**0.02**
Height (cm)	143.0 (24.0)	143.4 (28.3)	0.96
Height z-score	0.04 (2.77)	0.72 (1.04)	0.32
BMI (kg/m^2)	17.7 (3.1)	19.3 (3.9)	0.08
BMI z-score	-0.16 (0.89)	0.27 (1.03)	0.08

Values are mean (SD) for continuous variables or [n (%)] for categorical variables.
p-values are derived by the Student's independent samples *t*-test.
aIMT = aortic intima-media thickness; BMI = body mass index.

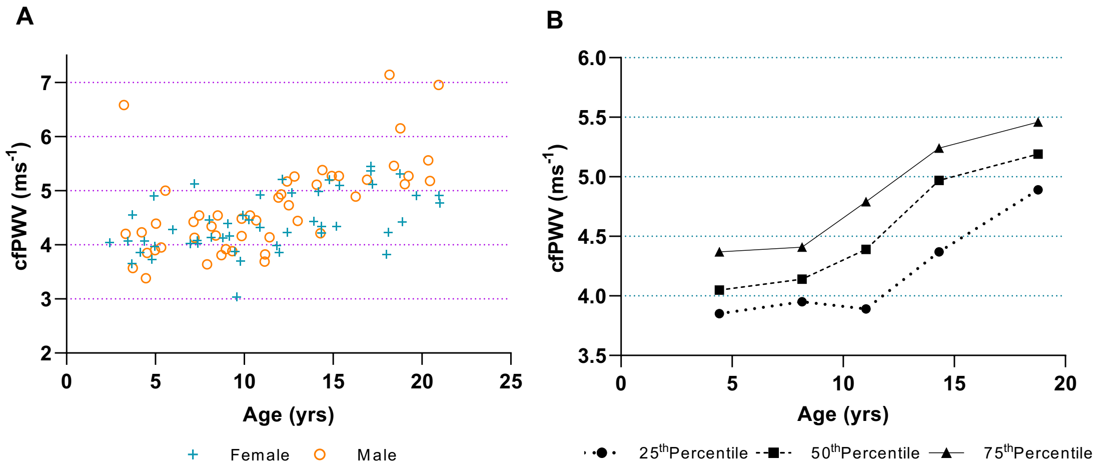

Supplementary Figure 1. Carotid-femoral pulse wave velocity (cfPWV) was measured via a validated cuff-based device in a prospective cohort of healthy males and females aged 2 - 20 years. (A) Scatter plot displaying age and cfPWV. (B) 25th, 50th and 75th percentiles of cfPWV according to median age.

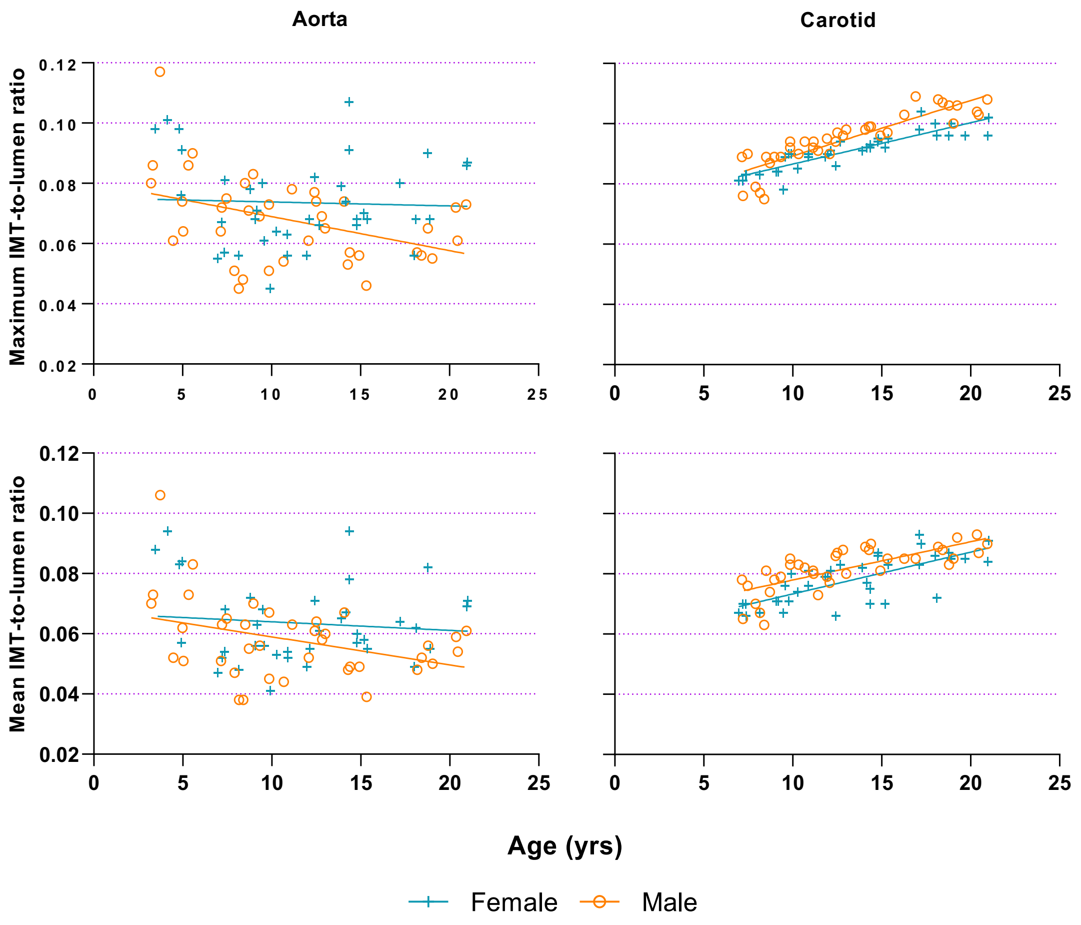

Supplementary Figure 2. Scatter plot displaying age trend of maximum and mean aortic intima-media thickness (aIMT) and carotid intima-media thickness (cIMT) to lumen ratio for males and females during childhood and adolescence. IMT-to-lumen ratio was calculated as IMT divided by vessel diameter at end-diastole. aIMT-to-lumen ratio was assessed in children aged 2 - 20; cIMT-to-lumen ratio was assessed in the subgroup of children aged 6.5 – 20.
